# Correlation between neutrophil gelatinase phase lipocalin and cerebral small vessel disease

**DOI:** 10.3389/fneur.2023.1177479

**Published:** 2023-07-14

**Authors:** Ying-hao Yang, Shan-shan Li, Yun-chao Wang, Lu-lu Yu, Hang-hang Zhu, Jing-hao Wu, Wen-kai Yu, Lu An, Wen-xin Yuan, Yan Ji, Yu-ming Xu, Yuan Gao, Yu-sheng Li

**Affiliations:** Department of Neurology, The First Affiliated Hospital of Zhengzhou University, Zhengzhou, Henan, China

**Keywords:** cerebral small vessel disease, inflammation, neuroimaging, total CSVD burden score, risk factors

## Abstract

**Background:**

Cerebral small vessel disease (CSVD) is common in the elderly population. Neutrophil gelatinase-associated lipocalin (NGAL) is closely related to cardiovascular and cerebrovascular diseases. NGAL causes pathological changes, such as damage to the vascular endothelium, by causing inflammation, which results in other related diseases. The purpose of this study was to investigate whether serum NGAL levels could predict disease severity in patients with CSVD.

**Methods:**

The patients with CSVD who visited the Department of Neurology at the First Affiliated Hospital of Zhengzhou University between January 2018 and June 2022 were prospectively included. The total CSVD burden score was calculated using whole-brain magnetic resonance imaging (MRI), and the patients were divided into a mild group (total CSVD burden score < 2 points) and a severe group (total CSVD burden score ≥ 2 points). Age, sex, height, smoking and alcohol consumption history, medical history, and serological results of patients were collected to perform the univariate analysis. Multivariate logistic regression was used to analyze the risk factors that affect CSVD severity. The multiple linear regression method was used to analyze which individual CSVD markers (periventricular white matter hyperintensities, deep white matter hyperintensities, lacune, and cerebral microbleed) play a role in the association between total CSVD burden score and NGAL.

**Results:**

A total of 427 patients with CSVD (140 in the mild group and 287 in the severe group) were included in the study. A multivariate logistic regression analysis showed that the following factors were significantly associated with CSVD severity: male sex [odds ratio(OR), 1.912; 95% confidence interval (CI), 1.150–3.179], age (OR, 1.046; 95% CI, 1.022–1.070), history of cerebrovascular disease (OR, 3.050; 95% CI, 1.764–5.274), serum NGAL level (OR, 1.005; 95% CI, 1.002–1.008), and diabetes (OR, 2.593; 95% CI, 1.424–4.722). A multivariate linear regression shows that periventricular white matter hyperintensities and cerebral microbleed are associated with serum NGAL concentrations (*P* < 0.05).

**Conclusion:**

Serum NGAL level is closely related to CSVD severity and is a risk factor for the burden of CSVD brain damage. Serum NGAL has high specificity in reflecting the severity of CSVD.

## 1. Introduction

CSVD refers to a group of clinical, cognitive, imaging, and pathological manifestations caused by structural and functional changes in small vessels, including cerebral small arteries and their distal branches, arterioles, capillaries, small venules, and venules (1, 2). CSVD can cause many types of vascular injury, which should be treated as a whole-brain disease (1, 2). The main imaging features of CSVD include recent small subcortical infarcts, presumed vascular-origin lacunae, presumed vascular-origin white matter hyperintensity (WMH), enlarged perivascular spaces (EPVSs), cerebral microbleed (CMB), and brain atrophy (3).

Although studies on the pathogenesis, etiology, and clinical manifestations of CSVD have made progress in recent years, clear diagnostic criteria and mechanisms of pathogenesis are lacking (4). Currently, CSVD is diagnosed mainly based on clinical and comprehensive imaging findings. The total CSVD burden score, which is a composite assessment for CSVD based on the four imaging features (WMH, CMB, EPVSs, and lacunar infarcts) of CSVD, may be more suitable for evaluating the overall brain damage based on imaging characteristics in CSVD (5). The pathogenesis of CSVD is believed to be a diffuse endothelial injury, which leads to increased vascular permeability, damage to the vessel wall, impaired autoregulation, and lumen narrowing and occlusion in the late stage, thus triggering discrete focal parenchymal ischemia and infarction (6). Studies have found that neutrophil gelatinase-associated lipocalin (NGAL) and NGAL/matrix metalloproteinase (MMP)-9 complexes act as pro-inflammatory factors in cardiovascular diseases. They play a role in the inflammatory response, the integrity of the endothelium, vascular remodeling, and plaque vulnerability (7–9). Cardiovascular and cerebrovascular diseases have certain similarities, which are reflected in common risk factors and mechanisms of occurrence (10). We performed this study to explore the relationship between NGAL and CSVD. By collecting the clinical and imaging data of patients, we calculated the total CSVD burden score to evaluate the correlation between serum NGAL level and CSVD severity.

## 2. Materials and methods

### 2.1. Patient selection

(i) According to the inclusion and exclusion criteria, 427 patients who were diagnosed with CSVD and had clinical manifestations such as dizziness, headache, paresthesias, fatigue, memory loss, and ataxia in the Department of Neurology, The First Affiliated Hospital of Zhengzhou University, Henan, China from January 2018 to June 2022 were included. Two neurology physicians collected the patient demographics, clinical characteristics, and imaging data and assessed the total CSVD burden score in each patient.
(ii) Inclusion criteria: (a) age ≥ 18 years; (b) CSVD lesions on magnetic resonance imaging (MRI) of the head with any of the following: WMH, and Fazekas score ≥ 2; Fazekas score = 1 and at least two vascular risk factors (e.g., hypertension, hyperlipidemia, diabetes, obesity, current smoking, and previous vascular events other than stroke); or Fazekas score = 1 with lacunar lesions; (c) independence in daily life, modified Rankin Scale (mRS) ≤ 2; and (d) provided signed informed consent.
(iii) Exclusion criteria: (a) in acute cerebral infarction, hyperintense lesions on diffusion-weighted imaging and >20 mm in diameter; (b) acute intracerebral hemorrhage; (c) acute subarachnoid hemorrhage, history of cerebrovascular malformation or aneurysmal subarachnoid hemorrhage, or discovery of untreated aneurysm (>3 mm in diameter); (d) diagnosed neurodegenerative diseases, such as Alzheimer's disease (11) and Parkinson's disease (12); (e) clear white matter lesions of non-vascular origin, such as multiple sclerosis, white matter dysplasia in adults, and metabolic encephalopathy; (f) psychiatric illness, such as depression, bipolar disorder, and acute anxiety disorder, diagnosed according to the Diagnostic and Statistical Manual of Mental Disorders (DSM-5); (g) contraindications to MRI (such as claustrophobia); (h) severe organic diseases, such as malignancy, severe chronic heart failure, and severe chronic renal failure, with an expected survival time of < 5 years; (i) being unable to complete the follow-up due to geographical or other reasons; and (j) concurrent participation in other clinical trials.

This study was approved by the Ethics Committee of the First Affiliated Hospital of Zhengzhou University.

### 2.2. Brain MRI

The total CSVD burden score was calculated using whole-brain MRI. (i) lacunar infarcts (LIs) ≥ 1 (1 point); (ii) WMH: according to the Fazekas scale (13), if there are irregular paraventricular hyperintensities extending to the deep white matter with a Fazekas score of 3 and/or the deep white matter lesions have begun to fuse or have high fusion in a large area with a Fazekas score of 2 or 3. WMH was scored as 1; (iii) cerebral microbleed (CMB): microbleeds are defined as small (5 mm) round lesions on low-intensity gradient echo images in the cerebellum, brainstem, basal ganglia, white matter, or cortical-subcortical junction; 1 point is awarded for ≥1 cerebral microbleed (3); (iv) EPVSs: EPVSs in the basal ganglia (EPVS-BS) are associated with CSVD and can be classified according to the number of EPVSs: mild EPVSs, ≤ 10; moderate EPVSs, 11–25; and extensive EPVSs, >25. Moderate or severe (>10) EPVS-BS was scored as 1 (14, 15).

The total CSVD score was calculated according to these criteria, and the patients were divided into mild and severe groups based on a total CSVD score of < 2 points and ≥2 points (total score of four points), respectively.

### 2.3. Data collection

Age, sex, height, smoking and alcohol consumption history, medical history, and serological results of patients were collected. Median cubital venous blood was collected after fasting for 8 h to determine the plasma NGAL levels. On the day of blood sample collection, the serum was prepared by centrifugation of whole blood at 3,000 r·min^−1^ and a radius of 10 cm for 10 min, and the supernatant was aspirated to avoid hemolysis and stored in a refrigerator at −80°C. A Human NGAL ELISA kit was obtained from Wuhan Huamei Biological Engineering Co., Ltd. in China (CSB-E09408h), and all samples were run in a single batch. According to the manufacturer's instructions, plasma NGAL levels were detected using an enzyme-linked immunosorbent assay, and the samples were prevented from repeated freezing and thawing. One replicate well was set for each sample for repeated measurements, and the average value was used for statistical analysis to reduce experimental errors.

### 2.4. Statistical methods

SPSS statistical software (26.0) was used for data analysis, and the count data were expressed as frequencies. If the data were distributed normally, the independent sample *t*-test was used, otherwise, the Mann–Whitney *U*-test was used; when theoretical frequency (T) was ≥5, Pearson's chi-square test was used; when T was < 5 and T was ≥1, the continuity-adjusted chi-squared test was used; and when *T* < 1, Fisher's exact test was used. Risk factors that were significantly (*p* < 0.05) associated with CSVD severity in the univariate analysis were entered into multivariable analyses. Multivariate logistic regression analysis by the Forward LR method was performed with CSVD patients with the CSVD load score level as the dependent variable. Differences were considered statistically significant at a *P*-value of < 0.05. The area under the receiver-operating characteristic curve (AUC) was used to evaluate the predictive value of serum NGAL for the severity of CSVD. Youden index (the sum of sensitivity and specificity minus 1, Youden index = sensitivity + specificity – 1) indicates the total ability of the test method to find real patients and non-patients. After calculating the Youden index, we sorted it and picked the largest Youden index, which is the best cutoff point, and then, we obtained its corresponding threshold values of AUC.

We further grouped individual CSVD markers separately: the mild group (which does not affect the total load score) and the severe group (which affects the total load score), for example, periventricular white matter hyperintensities (PWMH) in the mild group (Fazekas score < 3) and the PWMH severe group (Fazekas score = 3). Multiple linear regression was used to analyze the effect of individual CSVD markers (periventricular white matter hyperintensity, deep white matter hyperintensity, perivascular space enlargement, lacunes, and cerebral microbleed) on the relationship between total CSVD burden score and the NGAL level.

## 3. Results

### 3.1. Baseline characteristics

A total of 427 patients with CSVD were included in the study (140 in the mild group and 287 in the severe group).

### 3.2. Univariate analysis

The univariate analysis included the following parameters: there were significant differences in age, sex, hypertension, diabetes, cerebrovascular history, blood glucose, serum NGAL levels, serum total cholesterol (TC) levels, serum low-density lipoprotein (LDL-C) levels, glomerular filtration rate, HbA1 (%) levels, and absolute neutrophil count (ANC) among the groups (*P* < 0.05). There were no significant differences in cardiovascular history, smoking, alcohol consumption, serum triglyceride (TG) levels, serum high-density lipoprotein (HDL-C) levels, and homocysteine between the groups (Table 1).

**Table 1:** Study population characteristics and their correlation with total CSVD burden.

**Variable**	**Severe group (*n* = 140)**	**Mild group (*n* = 287)**	***P*-value**
Age	64.05 ± 10.96	57.67 ± 11.14	< 0.001^*^
Sex/male	193 (67.2%)	72 (51.4%)	0.002^*^
Cerebrovascular disease	130 (45.9%)	28 (20.1%)	< 0.001^*^
Cardiovascular history	32 (11.2%)	13 (9.4%)	0.564
Hypertension	202 (70.4%)	81 (58.3%)	0.013^*^
Diabetes	102 (35.5%)	22 (15.8%)	< 0.001^*^
Smoking	70 (24.4%)	25 (18.0%)	0.137
Alcohol consumption history	38 (13.4%)	15 (10.8%)	0.442
NGAL	149.58 (103.77–220.78)	111.76 (81.41–140.39)	< 0.001^*^
Blood glucose	5.43 (4.77–6.90)	5.04 (4.70–5.69)	0.001^*^
TC	3.78 (3.00–4.48)	4.09 (3.48–4.92)	0.011^*^
TG	1.14 (0.85–1.62)	1.29 (0.97–1.70)	0.079
H-LDL	1.07 (0.87–1.30)	1.14 (0.91–1.34)	0.331
L-LDL	2.18 (1.67–2.86)	2.44 (1.89–3.25)	0.011^*^
GFR	91.76 (81.66–100.12)	96.43 (89.52–103.80)	< 0.001^*^
HCY	13.16 (10.81–16.83)	12.29 (10.46–16.43)	0.082
HbA1 (%)	6.04 (5.60–7.08)	5.8 (5.55–6.20)	0.001^*^
ANC	4.08 (3.22–5.35)	3.36 (2.62–4.22)	< 0.001^*^

NGAL, neutrophil gelatinase-associated lipocalin; TC, total cholesterol; TG, triglyceride; HDL, high-density lipoprotein cholesterol; LDL, low-density lipoprotein cholesterol; GFR, glomerular filtration rate; HCY, homocysteine; HbA1, glycosylated hemoglobin; ANC, absolute neutrophil count; ^※^*P* < 0.05.

### 3.3. Multivariate analysis

We performed a multivariate logistic regression analysis on the statistically significant risk factors after preliminary screening. The results revealed that the following were significant risk factors for the total CSVD burden score (*P* < 0.05): male sex [odds ratio (OR), 1.912; 95% confidence interval (CI), 1.150–3.179], age (OR, 1.046; 95% CI, 1.022–1.070), history of cerebrovascular disease (OR, 3.050; 95% CI, 1.764–5.274), serum NGAL level (OR, 1.005; 95% CI, 1.002–1.008), and diabetes (OR, 2.593; 95% CI, 1.424–4.722) (Table 2).

**Table 2:** Multiple regression analysis of clinical characteristics affecting the total CSVD burden score.

**Variable**	**Group**	***P***	**OR**	**OR (95% CI)**
Sex	Women^*^			
	Men	0.012	1.912	1.150–3.179
Age		< 0.001	1.046	1.022–1.070
Cerebrovascular disease	No^*^			
	Yes	< 0.001	3.050	1.764–5.274
NGAL		0.001	1.005	1.002–1.008
Diabetes	No^*^			
	Yes	0.002	2.593	1.424–4.722

NGAL, neutrophil gelatinase-associated lipocalin. ^*^Control.

### 3.4. Receiver-operating characteristic curve

**Figure 1:**
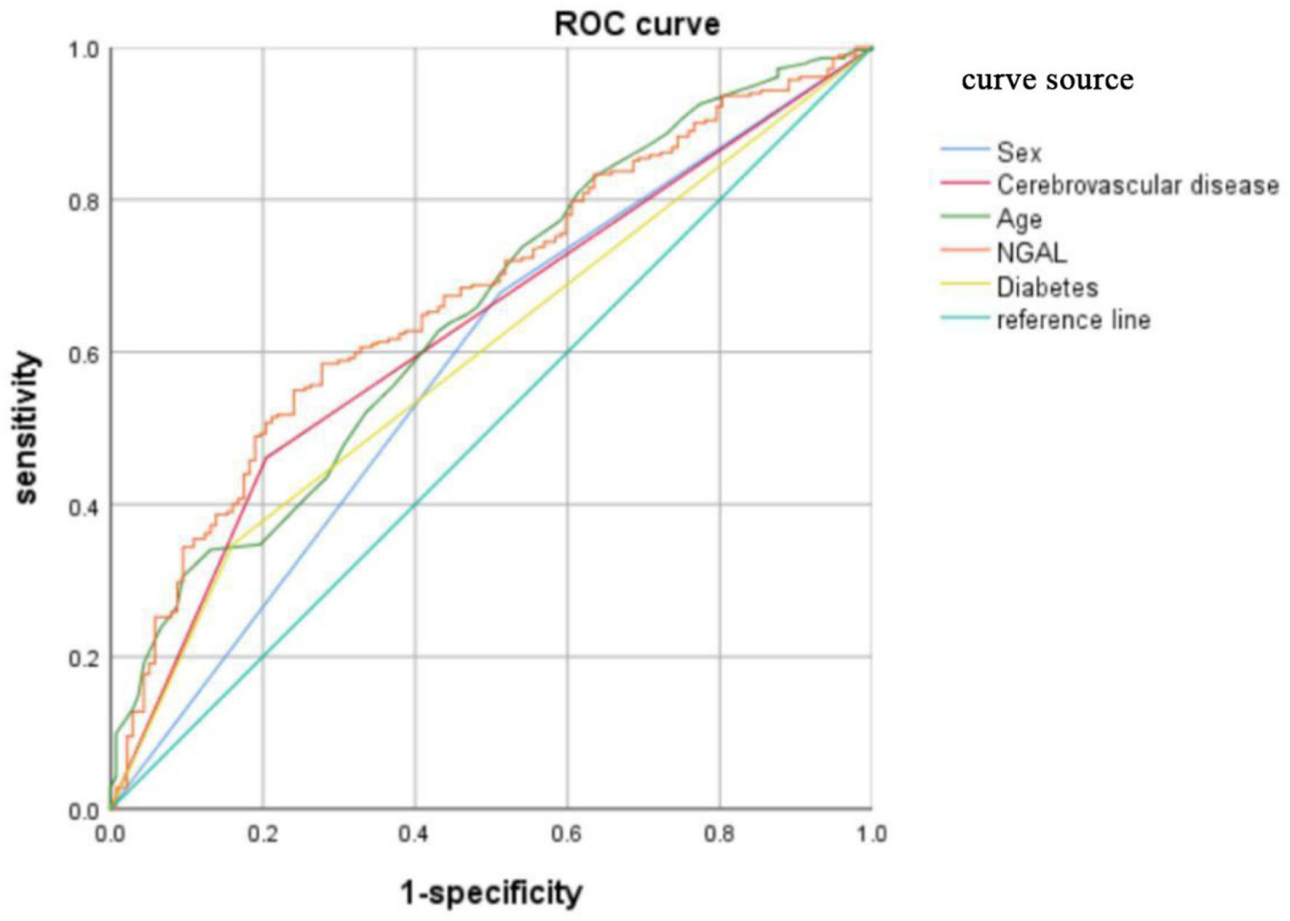
The receiver-operating characteristic curve.

The receiver-operating characteristic curve analysis revealed the following cutoffs: age of 70.5 years (sensitivity, 30.5%; specificity, 90.5%; AUC, 0.652), serum NGAL of 146.9 ng/ml (sensitivity, 55%; specificity, 75.9%; AUC, 0.674), history of cerebrovascular disease (sensitivity, 46.1%; specificity, 79.6%; AUC, 0.628), male sex (sensitivity, 67.7%; specificity, 48.9%; AUC, 0.583), and diabetes (sensitivity, 34.8%; specificity, 83.9%; AUC, 0.593). Serum NGAL has a higher area under the curve than traditional factors such as age and sex (Table 3). The receiver-operating characteristic curve is shown in Figure 1.

**Table 3:** ROC curve analysis.

**Index**	**Threshold**	**Sensitivity (%)**	**Specificity (%)**	**Area under the curve**
Age	≥70.5	30.5	90.5	0.652
NGAL	≥146.9	55.0	75.9	0.674
Cerebrovascular disease	Yes	46.1	79.6	0.628
Sex	Male	67.7	48.9	0.583
Diabetes	Yes	34.8	83.9	0.593

NGAL, neutrophil gelatinase-associated lipocalin.

### 3.5. Multiple linear regression

We analyzed the effect of individual SVD markers on the total CSVD burden score (Table 4).

**Table 4:** Multiple linear regression results.

**Marker**	**Beta**	***P***
PWMH	0.137	0.019
DWMH	0.044	0.455
CMB	0.126	0.029
Lacune	0.011	0.845

PWMH, periventricular white matter hyperintensities; DWMH, deep white matter hyperintensities; CMB, cerebral microbleed.

## 4. Discussion

In this study, we analyzed 427 patients with CSVD, including 140 in the mild group and 287 in the severe group, and found that the serum NGAL level was a risk factor for CSVD severity. Furthermore, serum NGAL level has different effects on individual SVD markers, especially between PWMH and DWMH.

CSVD is a dynamic disorder of the whole brain, and abnormal neurovascular unit (NVU) function plays an important role in its pathogenesis (16). The NVU is composed of neurons, astrocytes, vascular endothelial cells (EC), pericytes, and vascular smooth muscle cells that interact to regulate the fluid and nutrient entry into the interstitium, regulate cerebral blood flow, maintain and repair myelin sheaths, and scavenge metabolites for normal cellular function (1, 17, 18). Changes in the structure or function of the NVU can lead to CSVD. Common mechanisms for the destruction of the NVU include chronic cerebral ischemia and hypoperfusion (19), endothelial dysfunction (20), blood–brain barrier (BBB) disruption (21), interstitial fluid reflux disorders, inflammatory responses (22), and genetic factors (23).

### 4.1. Inflammatory responses and NGAL

Growing evidence suggests that inflammatory cytokines are associated with an increased risk of stroke, cardiovascular disease, and dementia (24–26). Additionally, the study by Gu et al. (27) and other studies reported that inflammatory biomarkers may play an active role in cerebrovascular injury.

NGAL is also known as ferritin or lipocalin 2 (28). The levels of NGAL in the brain originate not only from the brain but also from the blood (29). NGAL has been found to promote pro-inflammatory activation of glial cells and might enhance the infiltration of neutrophils and macrophages into the brain in certain conditions (30). NGAL can also promote the production of cytokines [such as interleukin (IL)-6, IL-8, and monocyte chemoattractant protein (MCP)-1] (7). The pro-inflammatory effect of NGAL plays an important role in the development of CSVD (31).

### 4.2. Blood-brain barrier disruption and NGAL

MMP-9 is a member of the zinc metalloprotease (ZMP) family. MMP-9 can destroy the BBB to allow macromolecules, such as fibrin, to enter the brain tissue, causing cerebral edema and structural damage (32). NGAL can form a heterodimer with the metalloprotease MMP-9 (NGAL/MMP-9) with stable biological activity (33). NGAL/MMP-9 prevents MMP-9 degradation and prolongs its activity by resisting the inhibitory effects of the tissue inhibitor of matrix metalloproteinase (TIMP) on MMP-9 (8).

A study found that NGAL can reduce the levels of the tight junction proteins claudin-5 and zonula occludens-1 in cultured brain endothelial cells (34). NGAL increases the BBB permeability, which can subsequently increase the leakage from blood vessels and macromolecules such as albumin and enter the brain parenchyma, thus aggravating CSVD (35).

### 4.3. Chronic cerebral ischemia, hypoperfusion, and NGAL

It is generally believed that NGAL, NGAL/MMP-9 complex, and cardiovascular disease, especially coronary heart disease and heart failure, are closely related (36, 37). The NGAL/MMP-9 complex allows leukocytes and cytokines to invade the intima and promotes the development of atherosclerosis (38). NGAL can also promote fibroblast hyperproliferation after myocardial infarction, thus resulting in interstitial myocardial fibrosis, increased left ventricular remodeling, and consequent heart failure (39). To our knowledge, cerebrovascular and cardiovascular diseases are similar in pathogenesis to some extent. We raised the hypothesis that NGAL may cause CSVD via the thickening of the vessel wall and narrowing of the lumen, leading to long-term chronic hypoperfusion of the cerebral. However, we still need more experiments to verify.

### 4.4. Endothelial dysfunction and NGAL

Endothelial dysfunction is commonly defined as a reduction in the bioavailability of nitric oxide (NO) in an EC (40). NGAL may disrupt the nitric oxide (NO) signaling pathway to reduce NO production (41). Dysregulation of NO leads to excessive vasoconstriction that affects cerebral blood flow (hypoperfusion), and long-term chronic cerebral ischemia increases the burden of CSVD (42, 43).

From the above analysis, it appears that the mechanism of NGAL causing the progression of CSVD is not independent but rather the overlapping of various mechanisms. These results also imply that we should pay more attention to NGAL.

A previous study reported that age is an important risk factor for CSVD (44), and the incidence of CSVD is highly age-related and does not vary by sex, ethnicity, or geography (45). Recent research showed that estrogen can promote cerebral blood flow, which is beneficial to nerve repair. Low serum estrogen levels can reduce cerebral blood flow and weaken neural repair mechanisms, which increases the risk of CSVD (46). A recent Mendelian randomization study strongly suggests a causal relationship between diabetes and CSVD, in particular with lacunar stroke and WMH (47). This is consistent with our conclusions.

In both ischemic and hemorrhagic stroke conditions, the most important etiopathogenesis is vascular damage. Occluded or ruptured cerebral blood vessels can cause inflammation, which in turn can be involved in stroke progression (48, 49). Stroke and CSVD have similar characteristics in cerebrovascular injury. A series of changes in the inflammation caused by stroke may lead to CSVD or accelerate the progression of CSVD.

However, several potential limitations should be acknowledged in the present study. First, it is a single-center study, and the sample size included in this study is relatively small. Second, according to the study results, the effect size of the NGAL level is low (OR, 1.005; 95% CI, 1.002–1.008). We did not include EPVS in the multiple linear regressions because of the low proportion of EPVS in our sample. Therefore, we need more experiments and large samples of data to verify. In our study, although the serum NGAL level is a non-traditional risk factor, indicating the degree of whole-brain small vessel damage in CSVD, sensitivity is not very high, which means that patients with cerebral small vessel disease who belonged to the severe group may be missed and diagnosed as a mild group by only using NGAL. However, we generally do not judge a certain disease based on only one biomarker in clinical trials. The combination of imaging and biomarkers is more beneficial to the diagnosis of CSVD.

CSVD is diagnosed mainly based on clinical and comprehensive imaging findings. However, the high cost, equipment requirements, and subjective judgment report are also limitations of imaging. The serum NGAL reflects the disease status of CSVD, which can give hints to clinicians and is more convenient and economical. Moreover, the combination of clinical, imaging, and NGAL may be more beneficial to the diagnosis of CSVD and the prediction of severity of CSVD. Our study is also conducive to the exploration and understanding of the mechanism of CSVD.

## 5. Conclusion

NGAL was originally used to evaluate renal damage. Recently, much attention has been paid to its role in cardiovascular diseases. However, there are few clinical studies on its role in cerebrovascular diseases, especially CSVD. This study fills this gap in the literature. We found that age, sex, history of stroke, diabetes, and serum NGAL level were closely related to the severity of CSVD and were risk factors for the overall brain damage burden in CSVD. We need to pay attention to NGAL and further explore the role of NGAL in CSVD in the future.

## Data availability statement

The original contributions presented in the study are included in the article/supplementary material, further inquiries can be directed to the corresponding authors.

## Ethics statement

The studies involving human participants were reviewed and approved by the Ethics Committee of the First Affiliated Hospital of Zhengzhou University (2021-KY-1059-002). The patients/participants provided their written informed consent to participate in this study.

## Author contributions

Y-mX and Y-sL: conception, design, and administrative support. Y-hY and Y-cW: provision of study materials or patients. S-sL, L-lY, and H-hZ: collection and assembly of data. J-hW, W-kY, LA, W-xY, YG, and YJ: data analysis and interpretation. All authors: manuscript writing and final approval of the manuscript.
